# A new type of flexible CP12 protein in the marine diatom *Thalassiosira pseudonana*

**DOI:** 10.1186/s12964-021-00718-x

**Published:** 2021-03-24

**Authors:** Hui Shao, Wenmin Huang, Luisana Avilan, Véronique Receveur-Bréchot, Carine Puppo, Rémy Puppo, Régine Lebrun, Brigitte Gontero, Hélène Launay

**Affiliations:** 1 CNRS, BIP UMR 7281, Aix Marseille Univ, 31 Chemin Joseph Aiguier, 13402 Marseille Cedex 20, France; 2 Key Laboratory of Aquatic Botany and Watershed Ecology, Wuhan Botanical Garden, Center of Plant Ecology, Core Botanical Gardens, Chinese Academy of Sciences, Wuhan, 430074 China; 3 Centre for Enzyme Innovation, School of Biological Sciences, Institute of Biological and Biomedical Sciences, University of Portsmouth, Portsmouth, PO1 2DY UK; 4 CNRS FR 3479, Plate-Forme Protéomique de L’Institut de Microbiologie de La Méditerranée (IMM), Aix Marseille Univ, 13009 Marseille, France

**Keywords:** Coiled coil, Diatom, Intrinsically disordered protein IDP, Nuclear magnetic resonance, Photosynthesis, Small angle X-ray scattering

## Abstract

**Background:**

CP12 is a small chloroplast protein that is widespread in various photosynthetic organisms and is an actor of the redox signaling pathway involved in the regulation of the Calvin Benson Bassham (CBB) cycle. The gene encoding this protein is conserved in many diatoms, but the protein has been overlooked in these organisms, despite their ecological importance and their complex and still enigmatic evolutionary background.

**Methods:**

A combination of biochemical, bioinformatics and biophysical methods including electrospray ionization-mass spectrometry, circular dichroism, nuclear magnetic resonance spectroscopy and small X ray scattering, was used to characterize a diatom CP12.

**Results:**

Here, we demonstrate that CP12 is expressed in the marine diatom *Thalassiosira pseudonana* constitutively in dark-treated and in continuous light-treated cells as well as in all growth phases. This CP12 similarly to its homologues in other species has some features of intrinsically disorder protein family: it behaves abnormally under gel electrophoresis and size exclusion chromatography, has a high net charge and a bias amino acid composition. By contrast, unlike other known CP12 proteins that are monomers, this protein is a dimer as suggested by native electrospray ionization-mass spectrometry and small angle X-ray scattering. In addition, small angle X-ray scattering revealed that this CP12 is an elongated cylinder with kinks. Circular dichroism spectra indicated that CP12 has a high content of α-helices, and nuclear magnetic resonance spectroscopy suggested that these helices are unstable and dynamic within a millisecond timescale. Together with in silico predictions, these results suggest that *T. pseudonana* CP12 has both coiled coil and disordered regions.

**Conclusions:**

These findings bring new insights into the large family of dynamic proteins containing disordered regions, thus increasing the diversity of known CP12 proteins. As it is a protein that is more abundant in many stresses, it is not devoted to one metabolism and in particular, it is not specific to carbon metabolism. This raises questions about the role of this protein in addition to the well-established regulation of the CBB cycle.

Choregraphy of metabolism by CP12 proteins in Viridiplantae and Heterokonta.

While the monomeric CP12 in Viridiplantae is involved in carbon assimilation, regulating phosphoribulokinase (PRK) and glyceraldehyde-3-phosphate dehydrogenase (GAPDH) through the formation of a ternary complex, in Heterokonta studied so far, the dimeric CP12 is associated with Ferredoxin-NADP reductase (FNR) and GAPDH. The Viridiplantae CP12 can bind metal ions and can be a chaperone, the Heterokonta CP12 is more abundant in all stresses (C, N, Si, P limited conditions) and is not specific to a metabolism.

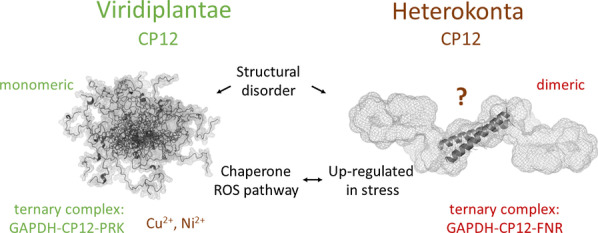

**Video Abstract**

**Supplementary Information:**

The online version contains supplementary material available at 10.1186/s12964-021-00718-x.

## Introduction

Chloroplast protein of 12 kDa (CP12) is a small nuclear encoded protein of about 80 amino acid residues, originally described by Pohlmeyer et al*.* [1] that occurs in many photosynthetic organisms [2, 3] and in Plantae and cyanobacteria is associated to the redox signaling pathway involved in the switch on/off of the light and dark metabolisms [4]. In higher plants, green and red algae, and cyanobacteria, it is associated with two enzymes, phosphoribulokinase (PRK) and glyceraldehyde 3-phosphate dehydrogenase (GAPDH) from the Calvin Benson Bassham (CBB) cycle that is responsible for CO_2_ assimilation [5]. This ternary complex has been well-studied and its structure has been recently solved using cryo-electron microscopy in the cyanobacterium *Synechococcus elongatus* [6] and using X-ray diffraction in the model higher plant *Arabidopsis thaliana* [7]. CP12 proteins from different organisms have some highly conserved regions such as a AWD_VEEL motif [2] and in most cases have a pair of cysteine residues at the C-terminus and/or a second pair at the N-terminus. Although cysteine residues are order-promoting amino-acids, and likely to structure the molecule, CP12 shares some physico-chemical properties with intrinsically disordered proteins (IDPs). In agreement with predictors of intrinsic disorder, CP12 from the green alga *Chlamydomonas reinhardtii* [8] and later from the angiosperm *A. thaliana* [9] were shown by circular dichroism (CD) and Nuclear Magnetic Resonance (NMR) to contain little regular secondary structure in solution [10–12].

These proteins have been extensively studied in these organisms and in *C. reinhardtii* it is a jack-of-all-trades that cannot only bind to PRK and GAPDH under its oxidized state, thereby downregulating their activity upon complex formation in the dark, but can also perform other functions [13]. For example, it can bind metal ions [14, 15], and can act as a specific chaperone-like for GAPDH [16]. In the tropical legume, *Stylosanthes guianensis*, higher expression of CP12 increases growth, plant height and photosynthesis rate [17]. Conversely, in the tobacco *Nicotiana tabacum* and in the mouse-ear cress *A. thaliana*, antisense suppression of CP12 reduces the rate of photosynthesis and increases the expression of proteins related to oxidative stress [18, 19]. Finally, in *A. thaliana*, one CP12 isoform is mainly expressed in non-photosynthetic tissues (roots and floral tissues) [20]. All these studies suggest that CP12 proteins have other functions beyond the dark downregulation of CBB enzymes and could act as possible signaling proteins coordinating multiple cell actions in response to environmental variables [21].

Little is known about CP12 in the diatoms, an ecologically important group of microalgae. Diatoms have a more complex evolutionary background than the other organisms mentioned above in which CP12 has been studied, and the regulation of their CBB enzymes is not fully understood [22]. The complex PRK-GAPDH-CP12 does not seem to be present [23–26] though there are some studies indicating a possible presence of CP12 in these organisms [23, 27]. For example, a CP12-like protein from *Thalassiosira pseudonana* has been shown to be expressed under stress conditions such as low CO_2_ [28] or low nutrients (nitrogen, phosphorus, silicon) [29]. Even though we do not know yet the function of CP12 from *T. pseudonana*, because it has homology with well-studied CP12s that allow organisms to respond to rapid changes, it is likely that this protein will have a similar role in diatoms that face fluctuating environments. The aim of this manuscript was therefore to characterize the structural properties of this protein from the marine diatom *T. pseudonana*, using both in silico and a range of biophysical experimental approaches.

## Material and methods

### Diurnal expression of *T. pseudonana* CP12 in vivo

Cells from *T. pseudonana* (strain CCMP 1335 from https://ncma.bigelow.org/) were grown in F/2 + Si medium (http://www.ccap.ac.uk/) under continuous light (50 µmol photon m^−2^ s^−1^) in an incubator (Innova 4230, New Brunswick Scientific) at 19 °C and shaken at 100 rpm. Growth of *T. pseudonana* was monitored using the absorbance at 680 nm. When the cells reached the exponential phase, half of the culture was put in the dark, and half left in the light. After 24 h, cells were collected by centrifugation for 15 min at 3275 g, 19 °C with a Beckman Allegra X15R centrifuge, and resuspended in 15 mM tris(hydroxymethyl)aminomethane (Tris), 4 mM ethylenediaminetetraacetic acid (EDTA), pH 7.9 with 0.5  μg mL^−1^ protease inhibitors (Sigma). The cells were sonicated (Sonic Ruptor 250, on ice, 4 cycles, 1 min sonication and 1 min rest), then centrifuged at 16,000 g for 20 min at 4 °C and the supernatant collected. Protein amount was measured by the Bradford protein assay using bovine serum albumin as a standard (Bio-Rad). Proteins were loaded to 12% sodium dodecyl sulfate polyacrylamide gel electrophoresis (SDS-PAGE) that was stained with Coomassie Blue or immediately transferred onto a 0.45 μm nitrocellulose membrane (Thermo Fisher Scientific). The antibodies raised against recombinant His-tagged CP12 in rabbits were produced by Eurogentec (https://www.eurogentec.com/en/custom-antibodies). The membrane was incubated first with $\alpha$- *T. pseudonana* CP12 antibodies diluted 1: 10,000, then with goat anti-rabbit IgG horseradish peroxidase (HRP, Invitrogen) diluted 1: 10,000. Finally, the membrane was revealed with luminol-based substrate (Amersham Enhanced Chemiluminescence western blotting kit detection reagent) using ImageQuant LAS 4000 biomolecular imager (GE Healthcare).

### Expression of CP12 during different phases of growth

A preculture of *T. pseudonana* cells in F/2 + Si medium, under continuous light (50 µmol photon m^−2^ s^−1^), was first grown at 19 °C, shaken at 100 rpm under high CO_2_ concentration (20,000 ppm) to increase biomass. After five days, the pellet of a 40 mL aliquot of this pre-culture obtained by centrifugation at 3275 g for 15 min at 4 °C was washed and re-suspended in fresh F/2 + Si medium. This was performed three times. These cells were then inoculated into fresh F/2 + Si medium at an initial absorbance at 680 nm of 0.2 (pathway length 1 cm) and grown under air-concentration of CO_2_ (400 ppm). Growth of *T. pseudonana* was monitored using the absorbance at 680 nm. Every day, a volume of the culture was collected that was normalized to obtain 30 μg of total proteins in cell extract. A pellet of cells was obtained after centrifugation for one minute at 3275 g and re-suspended into SDS-PAGE loading buffer containing 15 mM dithiothreitol. Cell lysis and protein denaturation were performed at 95 °C for 10 min. During the exponential phase and the beginning of the stationary phase, the expression of CP12 was monitored using western-blot analysis.

### In silico bioinformatic analysis

The subcellular localization of CP12 from five species of diatoms was predicted using a signal peptide predictor dedicated to diatoms, HECTAR (https://webtools.sb-roscoff.fr/) [30]. The signal peptides of CP12 from other organisms were predicted using ChloroP (http://www.cbs.dtu.dk/services/ChloroP/, [31]), SignalP (http://www.cbs.dtu.dk/services/SignalP/ [32]) and TargetP (http://www.cbs.dtu.dk/services/TargetP/ [33]). These enabled the N-terminus of the mature chloroplast CP12 proteins to be determined. Disordered regions were predicted using PONDR VL-XT (http://www.pondr.com/) [34], DisEMBL Remark-465 (http://dis.embl.de/) [35], IUPred2A (https://iupred2a.elte.hu/) [36] and s2D (http://www-mvsoftware.ch.cam.ac.uk/index.php/s2D) [37]. Coiled coil regions were predicted using Paircoil (http://cb.csail.mit.edu/cb/paircoil2/paircoil2-like.html), with a minimum search window of 21 residues [38]. Amino acid frequency in the algal and diatom CP12 was analyzed by composition profiler (http://www.cprofiler.org/cgi-bin/profiler.cgi) [39] against the Protein Data Bank (PDB) (https://www.rcsb.org/).

### Overexpression and purification of CP12 from *T. pseudonana*

Primers containing the *NdeI* and *BamHI* restriction sites were used to amplify and clone the CP12 gene in frame with the N-terminal histidine tag of the pET28a expression vector (Novagen) (forward primer 5′ CATATGGCTGCCATTGAAGCTGCTCT 3′ and reverse primer 5′ GGATCCCTAACGGGAACCAAGGGCC 3′). This plasmid was used to transform *Escherichia coli* BL21(DE3) pLysS. Freshly transformed bacteria were grown in 2YT medium with 50 µg/mL kanamycin and 34 µg/mL chloramphenicol at 37 °C until the absorbance at 600 nm reached 0.5–0.6 (1 cm pathlength). Cultures were cooled on ice for 30 min and then CP12 expression was induced with 1 mM isopropyl-β-d-1-thiogalactopyranoside (IPTG). Cells were cultured at 30 °C overnight in an incubator (Edmund Bühler GmbH, Fisher Bioblock Scientific), then centrifuged at 3275 g at 4 °C (Beckman Allegra X15R centrifuge). The pellets containing cells were re-suspended in 50 mM NaH_2_PO_4_/Na_2_HPO_4_, 300 mM NaCl, 10 mM imidazole, pH 8.0 (Ni-NTA buffer). Cells were then broken by sonication (Sonic Ruptor 250, 1 min sonication and 1 min on ice, 4 cycles) and centrifuged at 27,000 g for 20 min at 4 °C. The supernatant contained the recombinant histidine tagged CP12 (His-CP12) that was then purified by nickel ion affinity chromatography on Ni-NTA agarose column (Qiagen) (1.2 × 8 cm of resin). The column was equilibrated with Ni-NTA buffer. Contaminants were firstly washed out with 10 mM imidazole until the absorbance at 280 nm reached a minimum, then fractions were gradually eluted with an imidazole gradient (10–250 mM imidazole, 2 × 45 mL). Proteins elution was followed by absorbance at 280 nm. His-tagged CP12 was eluted with 150 mM imidazole, and dialyzed with 10 mM sodium phosphate buffer, pH 7.4 then stored at − 20 °C. Size exclusion chromatography (SEC), electrospray ionization coupled to mass spectrometry (ESI-MS) and circular dichroism (CD) were performed on CP12 after His tag removal with thrombin (T4648, Sigma) (1 U for 100 µg of CP12) at room temperature for 18 h. The sample was concentrated using a 500 µL spin X-UF ultra centrifugal concentrator, Corning, 5 kDa cut-off. After His-tag removal, CP12 was stored at − 20 °C.

### Determination of redox state

The free thiol groups in CP12 were quantified using 5,5′-dithiobis-(2-nitrobenzoic acid) (DTNB) (Sigma-Aldrich). 2 µM CP12 was mixed with 50 µM DTNB in 10 mM phosphate, 2 mM EDTA at pH 8 and the absorbance followed at 412 nm. A control without the protein was recorded in parallel. Number of free SH groups was calculated from N = $\Delta OD/\upepsilon CL$ (N: number of thiol; $\upepsilon$: DTNB extinction coefficient, 14,150 M^−1^ cm^−1^; $C$: protein concentration in M; $L$: light pathlength in cm).

### Native electrospray ionization-mass spectrometry (ESI–MS)

Prior to native ESI–MS analysis, CP12 without the histidine tag was dialyzed against 200 mM ammonium acetate buffer (pH 8.0) using 5 kDa cut-off concentrator columns (Spin-X, Corning). Experiments at 5 µM of CP12 were carried out on an electrospray Q-ToF mass spectrometer (Synapt G1 HDMS, Waters) using NanoLockSpray ionisation source with borosilicate emitter (NanoES spray capillaries, Thermo Scientific). Optimized instrument parameters were as follows: source pressure 5.3 mbar, source temperature 20 °C, capillary voltage 1.8 kV, sampling cone voltage 180 V, extractor cone voltage 4 V, trap collision energy 30 V and transfer collision energy 20 V. Mass spectrometer was calibrated in positive mode from 1000 to 5000 m/z with CsI (1 mg/mL) just prior acquisition.

### Size exclusion chromatography

500 µL of CP12 after histidine tag removal, at 0.5 mM were loaded on a Hiload Superdex 200 (prep grade 26 mm × 600 mm), unless stated otherwise, equilibrated with 150 mM NaCl, 50 mM sodium phosphate buffer at pH 7.5. When mentioned, reducing agent (1 mM Tris(2-carboxyethyl)phosphine, TCEP) was added to the buffer. The column was calibrated with six globular proteins of different molecular mass: thyroglobulin, ferritin, alcohol dehydrogenase, conalbumin, ovalbumin and carbonic anhydrase. Throughout elution, the absorbance at 280 nm was monitored to determine the presence of proteins. The fractions were collected, concentrated (spin X-UF ultra centrifugal concentrator, Corning, 5 kDa cut-off) and stored at − 20 °C.

### Circular dichroism (CD)

10 µM CP12 without histidine tag in 50 mM sodium phosphate pH 6.5 was used to record CD spectra (Jasco 815CD spectrometer, 2 mm thick quartz cells) scanned from 190 to 260 nm at a speed of 10 nm/min (n = 3). The data were analyzed using Dichroweb using the CDSSTR, SELCON3 and CONTIN analysis programs, and the reference set 7 (http://dichroweb.cryst.bbk.ac.uk/html/home.shtml) [40, 41] and Bestsel (http://bestsel.elte.hu/index.php) [42].

### Nuclear magnetic resonance (NMR)

^15^N-labelled histidine tagged CP12 were produced using the enhanced M9 medium (protocol of the European Molecular Biology Laboratory, https://www.embl.de/pepcore/pepcore_services/protein_expression/ecoli/n15/index.html) and purified as described above. The final sample was buffer exchanged in 50 mM sodium phosphate pH 6.5, 50 mM NaCl, 10% D_2_O with traces of sodium trimethylsilylpropanesulfonate (DSS) and at a final protein concentration of 250 μM.

The data were recorded at 4 °C and 15 °C. Fast ^1^H-^15^N heteronuclear single quantum correlation (fHSQC [43]) spectra were recorded with a ^1^H acquisition time of 243 ms, ^15^N acquisition time of 42 ms and with 24 scans on a 600 MHz NMR spectrum equipped with a cryogenic probe (Bruker). Translational diffusion was measured using standard bi-polar stimulated echo experiment [44], with a diffusion delay (Δ) of 200 ms. Ten experiments were recorded in which the encoding and decoding pair of gradients are produced with squared 1.4 ms long gradients (δ) with strength (G) ranging from 2 to 98% of the maximum gradient strengths (5.1 G mm^−1^). The data were processed using nmrPipe [45], plotted using Sparky[46]. The diffusion coefficient (D) was calculated using Octave [47] from the integral of proton signals of the methyl side chains of the protein from 1.2 to 0.7 ppm. The linear dependency of the logarithm of the integral as a function of the gradient strength was used to determine D as follows:

$$ln\left(\frac{{I}_{G}}{{I}_{0}}\right)=D.\left[\Delta -\frac{\delta }{3}\right]{.\left(\delta .{\text{G}}.{\gamma }_{H}\right)}^{2}$$

where I_G_ is the integral as a function of the gradient strength, I_0_ is the integral in the absence of gradient, γ_H_ is the proton gyromagnetic ratio, and Δ, δ and G are defined above. The hydrodynamic radius associated with the diffusion coefficient was determined using the Stokes–Einstein equation:

$${r}_{H}= \frac{{k}_{B}. T}{6\pi .D.{\eta }_{(T)}}$$

where k_B_ is the Boltzmann constant, T the temperature in Kelvin and ${\eta }_{(T)}$ the viscosity.

### Size-exclusion chromatography coupled to small angle X-ray scattering (SEC-SAXS)

SEC-SAXS experiments were performed on SWING beamline at the SOLEIL synchrotron using the online HPLC size exclusion chromatography facilities [48]. The sample-to-detector (CCD Aviex) distance was set at 2 m, leading to scattering vectors (q = 4 π/λsinθ, where 2θ is the scattering angle and λ the wavelength, equal to 1.033 Å) ranging from 0.01 to 0.46 Å. 50 µL of His-tagged CP12 (11 mg/mL) were injected into a pre-equilibrated size exclusion chromatography column (Agilent Bio-SEC-3300 Å) upstream of the measurement capillary at a temperature of 15 °C. Frames of 990 ms with dead-time of 10 ms were recorded throughout the elution, with 100 frames recorded at the very first minutes of the elution to measure the buffer background. The protein concentration was monitored via the absorbance at 280 nm with an in situ spectrophotometer. The experiment was performed in 30 mM Tris, 50 mM NaCl, 2 mM EDTA, 1 mM TCEP, pH 7.5.

Data reduction to absolute units and solvent subtraction were performed using FOXTROT, a dedicated in-house application. The frames recorded during the elution peak were carefully compared with each other, and data corresponding to identical scattering profiles and radius of gyration (Rg) were averaged to increase the signal-to-noise ratio. Data analysis was performed using the ATSAS suite of software [49]. The Rg and forward scattering intensity I(0) were obtained via PRIMUS using the Guinier approximation up to q.Rg < 1.0, and the distance distribution function P(r) was obtained via GNOM. The molecular mass of CP12 was determined using the forward scattering intensity I(0) of the frame corresponding to the top of the peak, at the highest protein concentration as described in [50]. Ab initio models of 3D envelopes corresponding to the scattering curve were constructed using DAMMIF (10 runs) and GASBOR (5 runs) with P1 and P2 symmetry [49].

### 3D-modelling

3D-modelling of the coiled coil domain has been performed using Swiss-model (https://swissmodel.expasy.org/ [51]) on a sequence including 5 residues before and 6 residues after the predicted coiled coil sequence highlighted in bold (APIVD**SEYEAKVKSLSQMLTKTKAELDQVKALADDLKGVK**LASPSV), without any other input. The model was fitted manually in the shape determined by SAXS.

## Results

### Expression of a CP12 protein in *T. pseudonana*

**Fig. 1:**
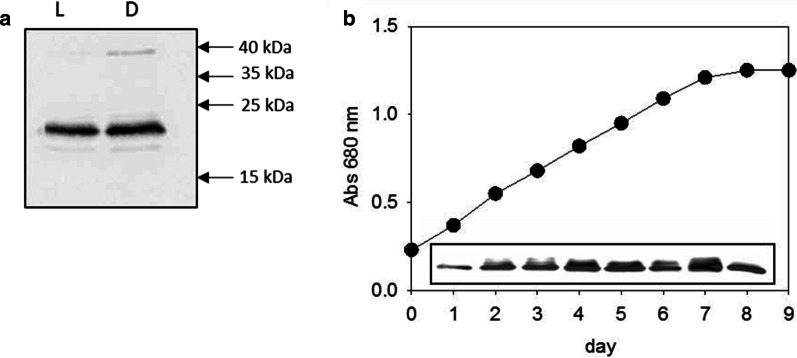
Western blot of CP12 expression. **a** Dark and light conditions. 20 µg crude extract from *T. pseudonana* cells grown under light (lane L) or dark (lane D). Molecular mass markers are shown on the right. After electrophoresis, the 12% SDS-gels are transferred onto nitrocellulose membranes and revealed with enhanced chemiluminescence with α- *T. pseudonana* CP12 antibodies 1:10,000. **b** Expression during the different phases of the growth curve. The growth of *T. pseudonana* in F/2 + Si medium, under continuous light and with air-concentration of CO_2_ (400 ppm) is followed by the absorbance at 680 nm. 30 μg of proteins from day 1 to 8 are loaded on SDS-PAGE gel. Proteins were transferred to a nitrocellulose membrane and revealed with antibodies raised against α- *T. pseudonana* CP12 antibodies

Chen et al. [29] reported a hypothetical protein of uncharacterized function that was overexpressed when *T. pseudonana* cells were limited by N, P or Si (identification: XP_002286772). This protein is identical to our previously identified CP12 protein that is expressed under low CO_2_ condition [28]. Our western-blot analysis indicates that CP12 protein was present in both dark-treated and light-treated *T. pseudonana* cells (Fig. 1a). Similarly, the expression level was stable during all phases of growth (Fig. 1b).

### In silico analysis of CP12 sequences

**Fig. 2:**
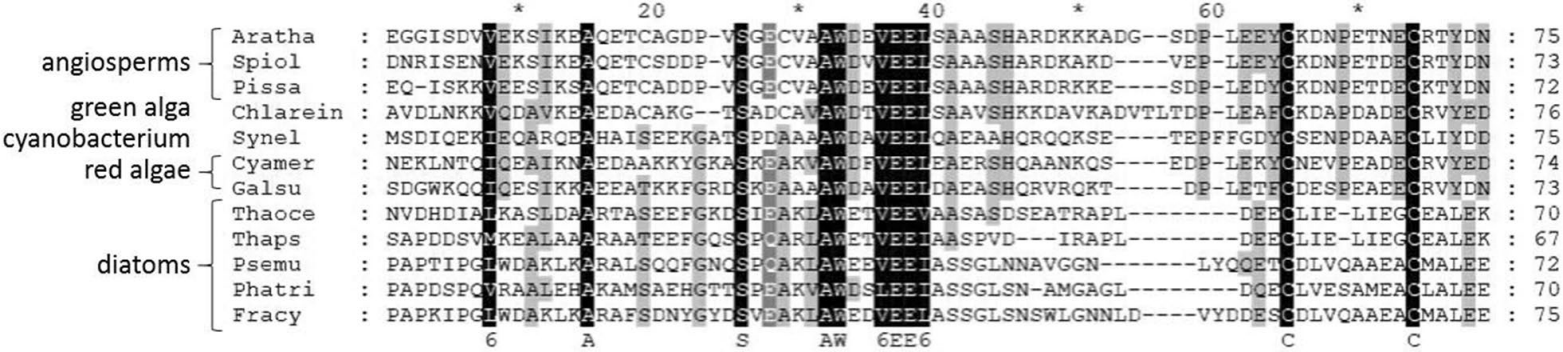
Alignment of CP12-like protein sequences from different species. Only partial sequences are shown. Species and protein IDs are the following: Aratha, *A. thaliana* (protein ID: NP_191800.1-NCBI, 131 aa); Spiol, *Spinacia oleracea* (protein ID: CAA96568.1-NCBI, 124 aa); Pissa, *Pisum Sativum* (protein ID: CAA96570.1-NCBI, 127 aa); Chlarein, *Chlamydomonas reinhardtii* (protein ID: XP_001694345.1-NCBI, 107 aa); Synel, *Synechococcus elongatus* (protein ID: Q6BBK3-UniProt, 75 aa); Cyamer, *Cyanidioschyzon merolae* (protein ID: BAM82525.1-NCBI, 122 aa); Galsu, *Galdieria sulphuraria* (protein ID: CAI34858.1-NCBI, 129 aa); Thaoce, *Thalassiosira oceanica* (protein ID: 95994-JGI, 165 aa); Thaps, *T. pseudonana* (protein ID: EED96413.1-NCBI, 197 aa); Psemu, *Pseudo-nitzschia multistriata* (protein ID: 292992-JGI, 256 aa); Phatri, *Phaeodactylum tricornutum* (protein ID: 44297-JGI, 329 aa); Fracy, *Fragilariopsis cylindrus* (protein ID: 244965-JGI, 234 aa). Alignments were performed with ClustalW, using MEGA7 software, and the figure was processed with GeneDoc (http://www.nrbsc.org/gfx/genedoc) with similar residues shaded using the conservation mode. The signal peptides are not included in the alignment

The sequence of this CP12 protein was aligned with other representative CP12 sequences from the angiosperms *A. thaliana*, *Pisum sativum* and *Spinacia oleracea*, the green alga *C. reinhardtii*, two red algae *Cyanidioschyzon merolae* and* Galdieria sulphuraria*, and the cyanobacterium *S. elongatus* (PCC 7942) (Fig. 2). Beside the highly conserved AWD_VEEL motif, one pair of cysteine residues at the C-terminus, another hallmark for CP12s, was also present. In contrast, the highly conserved proline positioned at the center of the 8-residues linker between these two cysteine residues is absent in CP12 from *T. pseudonana*. In the nuclear genome of other diatoms, *Phaeodactylum tricornutum*, *Thalassiosira oceanica*, *Fragilariopsis cylindrus* and *Pseudo-nitzschia multistriata*, a gene encoding a protein with high similarity with this CP12 protein was also found (Fig. 2). Furthermore, all these newly identified CP12 proteins from diatoms were predicted to be addressed to the chloroplast, in good agreement with the known localization of CP12 in other well-studied organisms.

### In silico characterization of the propensity of disorder

**Fig. 3:**
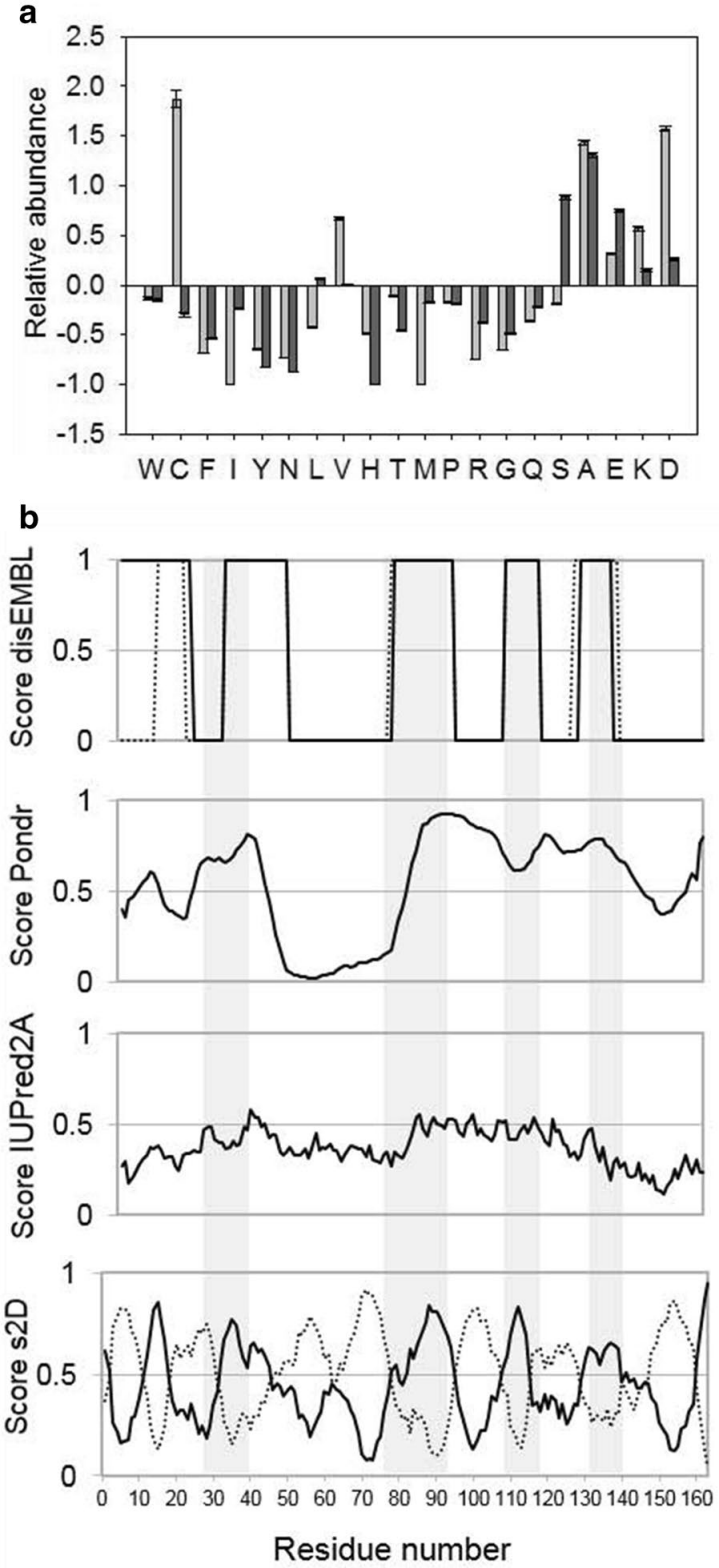
In silico analysis of CP12. **a** Amino acid composition of CP12s. The abundance of amino acids of both *T. pseudonana* CP12 (black bars) and *C. reinhardtii* CP12 (grey bars) were compared against proteins from the PDB database using Composition Profiler (http://www.cprofiler.org/cgi-bin/profiler.cgi). **b** Prediction of disordered region. The sequence on the x-axis is numbered according to the protein without tag and starting at AAIEAA. The residues propensity to be in a disordered region is shown as a score varying from 0 to 1, and these are predicted using, from top to bottom: disEMBL (for hot loop, in continuous line and for coil, in dotted line), PONDR-FIT, IUPred2A and s2D (continuous line). For most residues, s2D also predicts a significant propensity to form α-helices shown with dotted line

Since CP12 proteins from other organisms are IDPs [10, 12, 52], we checked whether CP12 from *T. pseudonana* was likely to contain disordered regions. The amino acid composition of this CP12 and that from *C. reinhardtii*, a well-known IDP, were compared to globular proteins. In the sequences of CP12 from *C. reinhardtii* and *T. pseudonana*, alanine (A) and charged residues that promote disorder such as aspartate, glutamate and lysine residues (D, E and K), are more abundant than in globular structured proteins (Fig. 3a). In contrast, order-promoting residues such as tryptophan, phenylalanine and tyrosine residues (W, F and Y) are less abundant than in structured proteins. In CP12 from *T. pseudonana*, cysteine residues are less abundant (two cysteine residues) than in the green algal CP12 (four cysteine residues).

It has an isoelectric point of 4.5 like many proteins, and interestingly, a very high negative net charge − 14.6 strengthening the hypothesis that this protein contains disordered regions.

The disorder propensity of CP12 from *T. pseudonana* was predicted using several algorithms. PONDR predicted a long disordered segment between residues 81–146, whereas the other predictors were more stringent (Fig. 3b), in particular IUPred2A which predicts only a small number of disordered regions. Taking all predictions together, four regions of disorder were consensually predicted which encompass residues 30–42, 77–93, 108–117 and 129–139 (shaded residues in Fig. 3b). Apart from the disordered regions, the algorithm s2D predicts the other regions to have a high propensity to form helices.

### Experimental characterization of the propensity of disorder

Abnormal migration is often observed in SDS-PAGE for IDPs, with an apparent molecular mass higher than the theoretical one. The theoretical molecular mass of a monomer of CP12 from *T. pseudonana* is 17.6 kDa but it migrated as a protein of higher molecular mass of about 22 kDa in SDS-PAGE (Fig. 1a). This abnormal migration resembles that of CP12 from *C. reinhardtii* where the molecular mass of the monomer with a histidine tag is ~ 11 kDa but migrates at about 20 kDa [8].

### Oligomeric state of CP12

**Fig. 4:**
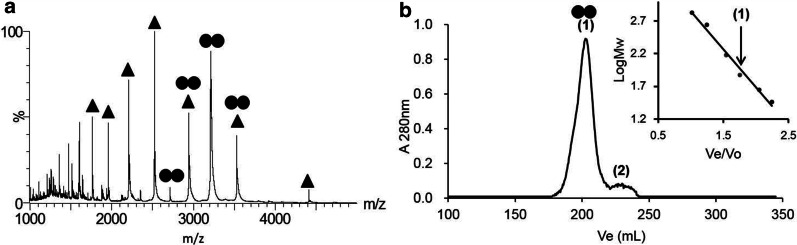
Oligomerization of CP12 revealed by native ESI–MS and size-exclusion chromatography. **a** ESI–MS spectrum of CP12. Signals labelled with a single triangle are assigned to monomeric CP12 (envelope of m/z 1765.2 to 4411.4 with charged states ranging from 10 to 4), and signals labelled with two circles are assigned to dimeric CP12 (envelope of m/z 2715.1 to 3529.4 with charged states ranging from 13 to 10). **b** Elution profile of CP12 on a S200 size-exclusion column. The single elution peak is assigned to dimeric CP12 (labelled with (1)). A small proportion of a smaller particle is assigned with the labelled (2) and is ascribed to impurities. The column was calibrated with proteins of known relative molecular mass (insert): Thyroglobulin (670 kDa), Ferritin (440 kDa), Alcohol dehydrogenase (150 kDa), Conalbumin (75 kDa), Ovalbumin (44 kDa), carbonic anhydrase (30 kDa). The linear fit (R^2^ = 0.98) indicates a dependency of the MW as a function of the elution volume (Ve) as: $MW= {10}^{-1.16\frac{Ve}{Vo}+4}$ with Vo being the elution volume of blue dextran (void volume of 110.3 mL). The elution volume of CP12 is indicated with an arrow

Oligomeric state of CP12 was investigated using native ESI–MS. Monomeric and dimeric forms were observed (Fig. 4a). Experimental deconvoluted values of 17,642.1 (± 0.5) Da and 35,285.3 (± 0.7) Da fit well with theoretical values for the monomeric and dimeric species, 17,641.8 Da and 35,283.6 Da respectively. Under size exclusion chromatography, a single and narrow elution peak was observed, even at very low protein concentration, suggesting a unique oligomeric state in solution (Fig. 4b). This oligomeric state is not due to a covalent bond through the cysteine residues, because size exclusion chromatography showed the same single elution peak both in the presence and in the absence of reducing agent (Additional file 1: Figure S1a). Upon addition of the Ellman’s reagent, DTNB, the absorbance at 412 nm immediately reached a value corresponding to two free thiol groups in recombinant CP12 from *T. pseudonana*. This result indicates that its two cysteine residues were not involved in either an intra- or inter-molecular disulfide bridge, even under atmospheric aerobic conditions. In addition, immediate titration of the thiol groups suggests that the cysteine residues are not buried and are readily accessible to the reagent. The two oligomeric forms observed by ESI–MS can be ascribed to a partial dissociation of the dimer during ionization of the molecular complex. This dissociation of the dimer further supports that it is not a covalent dimer. Finally, the discrepancy between the molecular mass determined by ESI–MS for the dimeric state and the size exclusion chromatography elution volume of CP12 (corresponding to an apparent molecular mass of 93 (± 4) kDa) suggests that CP12 is an extended dimer.

### CP12 is a dynamic extended dimer

**Fig. 5:**
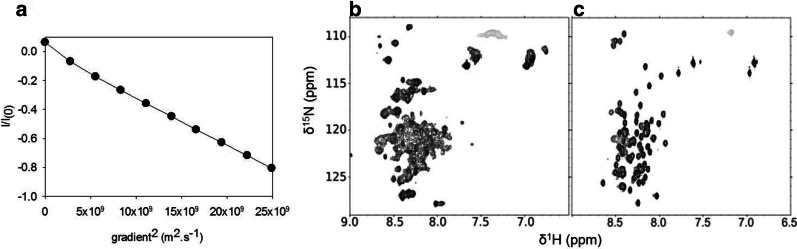
NMR spectrometric characterization of CP12: a dynamic dimer. **a** DOSY-NMR analysis of CP12, on the x-axis is the gradient strength and on the y axis is the normalized intensity on a ln scale (refer to Material and methods section). A linear dependency indicates a homogenous oligomerization of the protein. The linear fit indicates a diffusion coefficient of 3.8 (± 0.1) 10^–11^ m^2^ s^−1^, which relates to a hydrodynamic radius (Rh) of 3.4 (± 1) nm. **b** and **c** show the ^1^H-^15^N fHSQC spectra of CP12 from *T. pseudonana* and *C. reinhardtii* [10] respectively

The hydrodynamic size of CP12 in solution was confirmed by measuring the translational diffusion coefficient using DOSY-NMR (Fig. 5a). The translational diffusion coefficient was 3.8 (± 0.1) 10^−11^ m^2^ s^−1^ at 4 °C, which corresponds to a homogeneous species with a hydrodynamic radius (Rh) of 3.4 (± 1) nm. For a folded dimer of 35.3 kDa, the theoretical Rh is 2.75 nm, while for an unfolded dimer it is 5.94 nm [53]. Therefore, the experimental Rh of 3.4 nm represents an intermediate between that expected for a fully disordered and a fully ordered dimer and is also compatible with an extended dimer.

^1^H-^15^N fast-HSQC spectrum of CP12 presents some characteristic features of that of a disordered protein with the absence of ^1^H chemical shift dispersion (Fig. 5b). 34 resonances present narrow linewidths compatible with a purely disordered region. However, in contrast to true random-coil proteins such as CP12 from *C. reinhardtii* (Fig. 5c, [10]), the linewidths of the observed resonance varied from narrow (< 20 Hz) to broad (> 60 Hz), indicating the presence of an intermediate chemical exchange on the NMR timescale. This result suggests that reduced CP12 is highly dynamic on a range of timescale from ps to ms and does not possess a stable secondary structure.

**Fig. 6:**
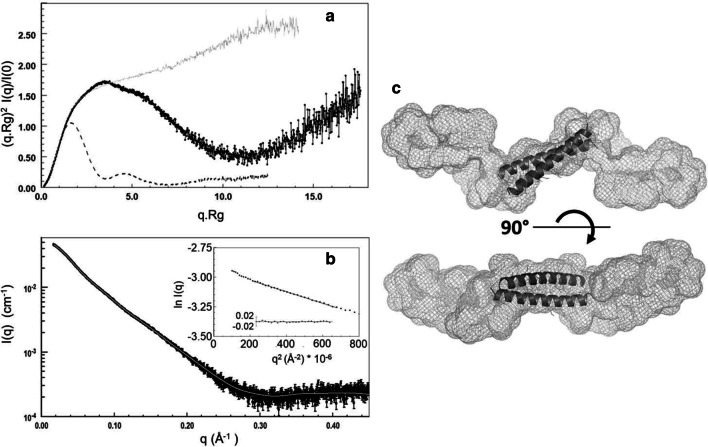
SAXS characterisation of CP12: an extended dimer. **a** Normalized and dimensionless Kratky plot. The data for *T. pseudonana* CP12 is shown in black, and can be compared with that for a fully disordered protein (*C. reinhardtii* reduced CP12 [10]), in grey, and that for a globular protein (*C. reinhardtii* GAPDH [11]) with dotted line. **b** Scattering intensity for CP12 (black) as a function of q (4π/λsinθ) overlaid with the DAMMIF fit (grey) related to the shape shown in **c**. The coiled coil structure calculated by Swiss-Model is inserted in the SAXS envelope (**c**). The insert in **b** shows the Guinier plot together with the residuals

We then performed SEC-SAXS experiment on CP12 to assess the overall structure and oligomeric state of disordered or partly disordered proteins (Fig. 6) [50]. The Guinier plot of the average spectrum was linear with a slight increase at very small q (q < 0.14 Å^−1^), indicating the presence of very small traces of aggregates that were not detected by the absorbance at 280 nm (Fig. 6b). The molecular mass inferred from the forward scattering intensity, independently of the shape of the protein, is 41.5 (± 3.0) kDa, confirming that CP12 is dimeric, as the theoretical molecular mass of dimeric histidine-tagged CP12 is 39 kDa. The inferred radius of gyration of CP12 was 38.2 (± 0.4) Å. The maximum dimension of the protein was 135 (± 5) Å, indicating that the protein is elongated. The normalized Kratky plot exhibited a large peak at (q.Rg) = 3.4 and (q.Rg)^2^.I(q)/I(0) = 1.7, followed by a decrease and then an increase of the curve, and is typical of a protein containing both globular folded domain(s) and disordered region(s) (Fig. 6a) [50]. Determination of the envelope of CP12 gave similar results using DAMMIF or GASBOR, applying a P1 or P2 symmetry, with excellent fits to the data (χ^2^ = 1.61–1.66 with DAMMIF, and 1.39–2.39 with GASBOR, Fig. 6b). The envelope is very elongated and forms a cylinder of ~ 20–25 Å diameter, with several kinks which may reflect the limits between different domains (Fig. 6c). This is also consistent with the presence of several disordered regions as previously mentioned (Fig. 3b).

### Secondary structure of CP12

**Fig. 7:**
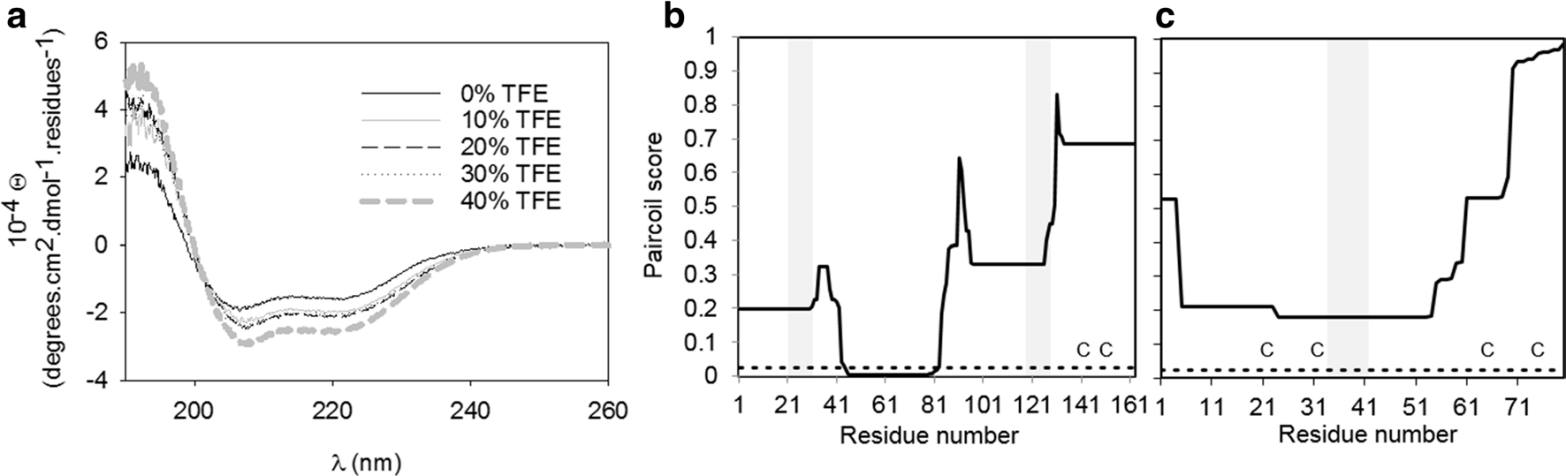
CD characterisation of CP12: high helical content and putative coiled coil arrangement. **a** CD spectra of 10 µM CP12 in 50 mM phosphate buffer pH 6.5 with increasing concentration of TFE. These spectra indicate a high proportion of helical structure, which increases further in the presence of TFE. **b**, **c** Result of the Paircoil predictor for the search of putative coiled coil motif for each *T. pseudonana* CP12 residues (**b**) and *C. reinhardtii* CP12 residues **(c)**. The coiled coil prediction is shown as a score varying from 0 to 1 (a score of 0 indicates a high probability to be in a coiled coil region and in contrast a score of 1, a low probability). The threshold for a region to have a high probability to form a coiled coil structure is indicated with a dotted line (score < 0.025). The AWD_VEEL regions are highlighted in grey, and the position of the cysteine residues are indicated with a C

Circular dichroism (CD) was used to determine the secondary structure of CP12. The CD spectrum had two minima at 208 and 222 nm (Fig. 7), and its deconvolution reveals a high content of α-helices (from 32 to 50%), plus 10% of β-sheets and from 27 to 48% of random coil (Additional file 1: Table S1). This result is consistent with the prediction of both disordered and helical regions using the s2D predictor. To investigate further the conformation of CP12, the solvent 2,2,2-trifluoroethanol (TFE), known to stabilize helical structures, was added. The CD spectra with increasing TFE concentrations showed an increase of the content of α-helices up to 79%, as revealed by lower values of ellipticity at 208 and 222 nm. We observed an isosbestic point at 201–202 nm, indicating the existence of two conformers in equilibrium (Fig. 7a). The presence of unstable helical structures in a dimeric and elongated protein is reminiscent of a coiled coil propensity, we thus used a predictor for coiled coil regions and found that the region encompassing residues 46–82 (Fig. 7b) has a very high propensity for coiled coil arrangement. Furthermore, 3-D modelling of this domain using Swiss-Model yielded a dimeric coiled coil structure which can be accommodated into the central region of the SAXS envelope (Fig. 6c). The propensity to form coiled coil structures might be a characteristic shared with other CP12 proteins, and at least CP12 from *C. reinhardtii* has a weak propensity to form coiled coil in the region encompassing the AWD_VEEL motif (Fig. 7c). These in silico predictions are consistent with our experimental data; in particular the SAXS results are consistent with a putative central coiled coil domain flanked by other elongated domains likely to be disordered and/or possibly containing short structural elements.

## Discussion

CP12 proteins from different organisms are highly diverse at the protein sequence level [2] but have some characteristic features such as the AWD_VEEL motif and often a pair of cysteine residues, at the C terminus, separated by eight residues encompassing a proline residue. The CP12 proteins from *C. reinhardtii*, *A. thaliana* and *S. elongatus* have been extensively studied and shown to belong to the IDP family. IDPs, or in other words ductile, dancing, malleable or flexible proteins [54], are common in various proteomes [55–60] and occupy a unique structural and functional niche in which function is directly linked to structural disorder [52, 61–63]. The absence of structure has been proposed to be a significant advantage for proteins whose functions are related to stress response [64], as e.g. in the oncologic context [65]. Furthermore, protein structural disorder is an asset with respect to fluctuating environmental conditions as faced by diatoms [52]. The dynamic structural properties of CP12 proteins could similarly confer them functional advantage, and indeed, some of their functions are related to oxidative stress response in higher plants [18, 19, 21].

*T. pseudonana* CP12 is indeed more expressed under all tested stress-related conditions (nutrient deprivation [29], low CO_2_ [28]), suggesting that CP12 is involved in a broad range of metabolisms. Only 37 proteins, including CP12, among 1111 proteins differentially expressed in three macronutrients deficient (Si, P, N) cells, are more abundant in all these stresses [29]. As such, CP12 can be considered as a stress protein, since stress proteins are a diverse group of proteins that are synthesized at increased levels under a variety of stressful stimuli, and have a protective effect against the stress. We speculate that its dynamical properties that we describe in this study are related to this function.

Besides, in this study, we show that the expression of CP12 from the diatom *T. pseudonana* is constitutively expressed under dark and light conditions, as well as during the growth, unlike CP12 from *A. thaliana* that is co-expressed with PRK and GAPDH in the light [66]. In contrast in this diatom, while CP12 is upregulated in stress conditions, GAPDH and PRK are downregulated [29], and this indicates that CP12 may have other functions in diatoms compared to viridiplantae.

Like CP12 from other organisms [8, 12, 55], CP12 from *T. pseudonana* is characterized by several features of IDPs, with a lower proportion of order-promoting residues and a higher proportion of charged residues than structured proteins. This high net charge and low overall hydrophobicity is concordant with the absence of rigid globular core as observed using NMR and with its high flexibility on the NMR-timescale. Another IDP characteristic is the ability to be involved in the formation of macromolecular complex, and this is the case for CP12 proteins from other organisms that bind to GAPDH and PRK [8, 9, 18, 67–69]. In contrast, the PRK-GAPDH-CP12 ternary complex has not been found in diatoms. This was attributed to the absence of cysteine residues on PRK in diatoms [25] but may also be the consequences of specific features of this CP12. Moreover, in a freshwater diatom, *Asterionella formosa*, GAPDH interacts with the ferredoxin-NADP reductase (FNR), from the primary phase of photosynthesis, and a small protein identified as a CP12 [70]. Diatom chloroplasts lack the oxidative pentose phosphate pathway, the main NADPH generating source in the dark. In the ternary complex GAPDH-CP12-FNR, GAPDH, a main NADPH consumer, is inhibited [23], thereby releasing the pressure on NADPH availability for other metabolic pathways.

While CP12 from *T. pseudonana* is predicted to contain at least a long disordered region, the proportion of α-helices in CP12 from *T. pseudonana* (50%) is much higher than in CP12 from *C. reinhardtii* [11] (30%) and in canonical IDPs. In addition, SEC-SAXS and native ESI–MS showed that CP12 from *T. pseudonana* is dimeric unlike CP12 from other species where it is monomeric in its free isolated state [10–12]. This dimeric state is homogeneous in solution as shown by SEC and DOSY-NMR, and is induced neither by formation of a disulfide bridge, nor by the presence of the His-tag (Additional file 1: Figure S1b). Dimeric oligomerization of very dynamic proteins is mediated by numerous but transient inter-molecular interactions, and this is a feature of coiled coil arrangement [71–73]. Indeed besides long disordered regions, CP12 from *T. pseudonana* is also predicted to contain a region that has a high probability to form coiled coils, and this is consistent with the extended hydrodynamic radius experimentally observed by gel filtration and DOSY-NMR, and with the global shape derived from the SAXS data (Fig. 6c). Coiled coil is an omnipresent protein fold, accounting for about 3–10% of all protein-coding regions across all genomes [74–76]. Proteins having coiled coils are highly dynamic and contribute to an ever-growing list of functional contexts [71–73] and coiled coils are one of the most ubiquitous protein–protein interaction motifs [75]. Although CP12 from *C. reinhardtii* is not predicted to form coiled coil structures with the same high probability as *T. pseudonana* CP12, they both share a propensity to form unstable helices [11]. Such highly dynamic and adaptative biophysical properties is a common feature of moonlighting proteins [13].

## Conclusion

The structural properties of CP12 from *T. pseudonana*, with putative dimeric coiled coil domain and disordered regions, suggest that it is not an alien as regard to other CP12s and might therefore have one-to-many functions. As the gene encoding this protein has also been found in other diatoms, it could be another facet of the enigmatic regulation of diatom metabolism [22, 26, 77]. These findings extend the context for dynamic and coiled coil proteins related to their functions in photosynthesis regulation and stress in diatoms.

## Supplementary Information

**Additional file 1.** **Supplementary Figure 1**. The oligomerisation of CP12 is independent of the His-tag and does not involve disulfide bond. a Normalised SEC profile of recombinant His-tagged CP12 with (red) and without (blue) 1mM TCEP recorded on a Superdex 200 Increase 10 mm x 300 mm. Under reducing condition, His-tagged CP12 was treated with 10 mM TCEP before injection onto the column. b Normalised SEC profile of recombinant CP12 before (blue) and after His-tag removal (red) by thrombin, recorded on an Agilent Bio-SEC-3 300 Å column. **Supplementary Table 1**: Percentage of α-helices, strand, turn and other type of secondary structures of CP12 (included unstructured) derived from the CD profiles using different deconvolution methods: Bestsel, Dichroweb using the CDSSTR, SELCON3 and CONTIN methods and the reference set 7.

## Data Availability

Not applicable.

## Abbreviations

CBB: Calvin-Benson-Bassham
CD: Circular dichroism
CP12: Chloroplast Protein of 12 kDa
DOSY-NMR: Diffusion ordered spectroscopy-nuclear magnetic resonance
DTNB: 5,5′-Dithiobis-(2-nitrobenzoic acid)
EDTA: Ethylenediaminetetraacetic acid
ESI–MS: Electrospray ionization-mass spectrometry
FNR: Ferredoxin-NADP reductase
GAPDH: Glyceraldehyde phosphate dehydrogenase
HSQC: Heteronuclear single quantum coherence
IDP: Intrinsically disordered protein
NMR: Nuclear magnetic resonance
PRK: Phosphoribulokinase
SAXS: Small angle X-ray scattering
SDS-PAGE: Sodium dodecyl sulfate polyacrylamide gel electrophoresis
SEC: Size exclusion chromatography
TCEP: Tris(2-carboxyethyl)phosphine
Tris: Tris(hydroxymethyl)aminomethane
