# Supplementary material for: A new type of flexible CP12 protein in the marine diatom Thalassiosira pseudonana
Source: Cell Commun Signal. 2021 Mar 24;19:38. doi: 10.1186/s12964-021-00718-x (PMC7992989; doi:10.1186/s12964-021-00718-x)
Supplement: Supplementary file 2 — Additional file 1. Supplementary Figure 1. The oligomerisation of CP12 is independent of the His-tag and does not involve disulfide bond. a Normalised SEC profile of recombinant His-tagged CP12 with (red) and without (blue) 1mM TCEP recorded on a Superdex 200 Increase 10 mm x 300 mm. Under reducing condition, His-tagged CP12 was treated with 10 mM TCEP before injection onto the column. b Normalised SEC profile of recombinant CP12 before (blue) and after His-tag removal (red) by thrombin, recorded on an Agilent Bio-SEC-3 300 Å column. Supplementary Table 1: Percentage of α-helices, strand, turn and other type of secondary structures of CP12 (included unstructured) derived from the CD profiles using different deconvolution methods: Bestsel, Dichroweb using the CDSSTR, SELCON3 and CONTIN methods and the reference set 7. [file 12964_2021_718_MOESM2_ESM.pdf]

Supplementary data

A new type of flexible CP12 protein in the marine diatom *Thalassiosira pseudonana*

[1]Hui Shao, [1,2]Wenmin Huang, [1,3]Luisana Avilan, [1]Véronique Receveur-Bréchot, [1]Carine Puppo, [4]Rémy Puppo, [4]Régine Lebrun, [1,*]Brigitte Gontero & [1,*]Hélène Launay

Supplementary Figure 1: The oligomerisation of CP12 is independent of the His-tag and does not involve disulfide bond. **a** Normalised SEC profile of recombinant His-tagged CP12 with (red) and without (blue) 1mM TCEP recorded on a Superdex 200 Increase 10 mm x 300 mm. Under reducing condition, His-tagged CP12 was treated with 10 mM TCEP before injection onto the column. **b** Normalised SEC profile of recombinant CP12 before (blue) and after His-tag removal (red) by thrombin, recorded on an Agilent Bio-SEC-3 300 Å column.

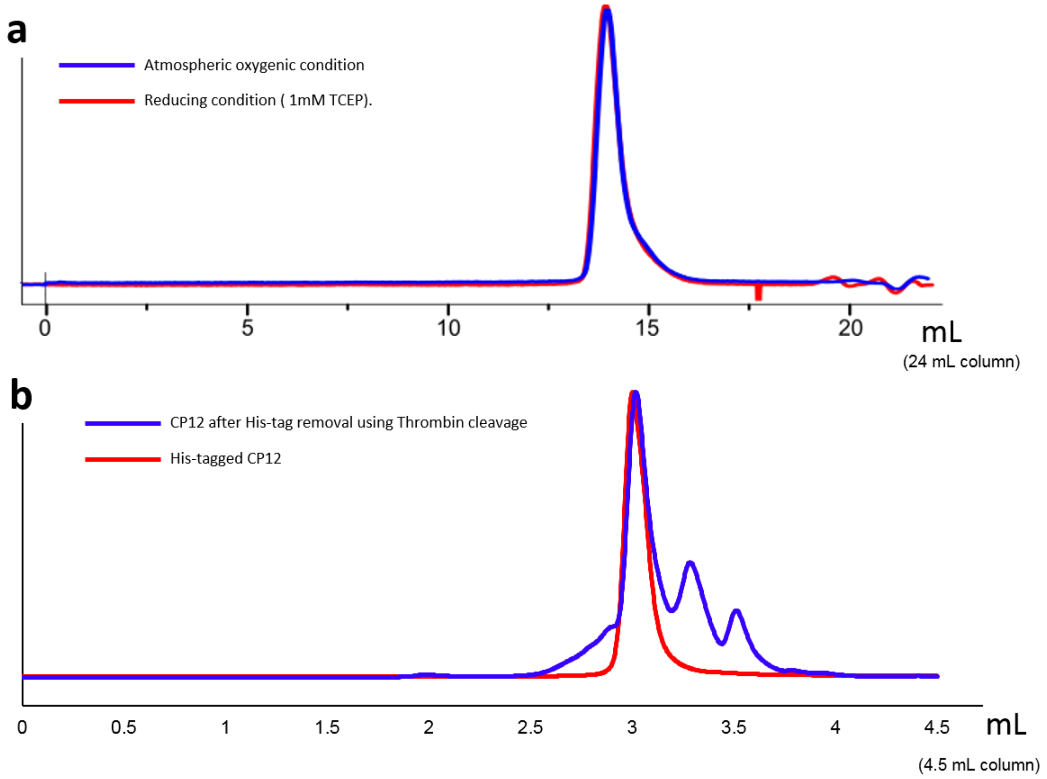

Supplementary Table 1: Percentage of α-helices, strand, turn and other type of secondary structures of CP12 (included unstructured) derived from the CD profiles using different

deconvolution methods: Bestsel, Dichroweb using the CDSSTR, SELCON3 and CONTIN methods and the reference set 7.

	**Bestsel**	**Dichroweb CDSSTR method**	**Dichroweb SELCON3 method**	**Dichroweb CONTIN method**
Helix (total)	32%	50%	44%	43%
Others /unordered	48.6%	27%	25%	33%
Strand	9.4%	10%	10%	7%
Turn	9.9%	14%	21%	17%
